# Effect of COVID-19 stressors on healthcare workers’ performance and attitude at Suez Canal university hospitals

**DOI:** 10.1186/s43045-021-00084-x

**Published:** 2021-01-26

**Authors:** Mohammed Goda Elbqry, Fatma Mohmed Elmansy, Abeer Ezzat Elsayed, Bassam Mansour, Ashraf Tantawy, Maged Bahi Eldin, Haydy Hassan Sayed

**Affiliations:** 1 Medical Surgical Nursing, Faculty of Nursing, Suez Canal University, Ismailia City, Egypt; 2 Medical Microbiology and Immunology, Faculty of Medicine, Suez Canal University, Ismailia City, Egypt; 3 Infectious and Endemic Diseases, Faculty of Medicine, Suez Canal University, Ismailia City, Egypt; 4 Psychiatric and Neurological Diseases, Faculty of Medicine, Suez Canal University, Ismailia City, Egypt; 5 Medical Psychiatric Department, Medical Academic Armed Force, Cairo, Egypt

**Keywords:** COVID-19 psychological stressors, Healthcare workers performance

## Abstract

**Background:**

Coronavirus disease 2019 is an emerging respiratory disease caused by a novel coronavirus effect on 10-20% of total healthcare workers and was first detected in December 2019 in Wuhan, China. This study was designed to assess effect of COVID-19 stressors on healthcare workers’ performance and attitude. A descriptive cross sectional research design was used. A convenient sample (all available healthcare workers) physicians “112,”, nurses “183,” pharmacists “31,” and laboratory technicians “38” was participated to conduct aim of the study. Utilize the study with two tools; online self-administrated questionnaire to assess level of knowledge, attitude, and infection control measures regarding coronavirus disease 2019 and COVID-19 stress scales to assess the varied stressors among healthcare workers.

**Results:**

More than three quarter of the studied participants had satisfactory level of knowledge and infection control measures. Approximately all of the studied participants had positive attitude regarding COVID-19. A total of 57.4% of the studied medical participants had moderate COVID-19 psychological stress levels, while 49.1% of the studied paramedical participants had moderate COVID-19 psychological stress levels. But less than one quarter had severe COVID-19 psychological stress levels. There is a significant correlation between COVID-19 psychological stressor levels and satisfactory level of knowledge among medical participants.

**Conclusion/implications for practice:**

Most of healthcare workers had satisfactory level of knowledge, infection control measures, and positive attitude regarding COVID-19. Most of them had moderate COVID-19 psychological stress levels.

## Background

The outbreak of a new coronavirus (COVID-19, formerly known as nCoV-2019) was first reported in Wuhan, China, since late December 2019. COVID-19 is an acute fatal disease that may cause progressive respiratory complications which end up with death [1, 2]. In April 2020, there was more than 1 million cases of infected patients all over 60 countries around the world. Based on the data from 72,314 cases, 14% had serious and 5% of the patients had critical conditions, with mortality rate of 2.3% [3]. At the national level, Egypt has recorded more than 500 cases and 60 deaths, while in the Suez Canal University Hospital, it is a local record; 10 nurse cases and 3 physicians have been recorded [4, 5].

Healthcare workers (HCWs) are exposed to multiple infectious diseases, which transmitted through the blood or other body fluids and/or airborne infectious [6]. HCWs are exposed to highest levels of risk when they are in direct contact with the patients [7] or while they care for patients or by exposure to patient biological samples or environment. Which make them worry of being infected and transmitting infection to family members and have negative effects on them (C [8].; D [9].). Stress and job burnout among the HCWs are more during a pandemic outbreak of an infectious disease [10].

Widespread infection and fatalities among the HCWs are causing social and mental pressures on them which have been reported previously for SARS and MERS and currently for the COVID-19 disease [11]. A conceptual framework for healthcare workers’ stress when caring for COVID-19 patients, including four variables (the worry of social isolation, the discomfort caused by the protective equipment, the difficulties and anxiety of infection control, and the workload of caring for patients) [11].

The lack of knowledge has been associated with higher infection rate [12]. Misunderstandings among HCWs have delayed controlling efforts to provide necessary treatment [13], led to the rapid spread of infection in hospitals, and put patients’ lives at risk. Knowledge can influence the perceptions of HCWs due to their past experiences and beliefs [14].

Several socio-demographic (e.g., gender, age, profession) and psychological variables (e.g., social support, self-efficacy) have been associated with increased level of stress, anxiety, depressive symptoms, and insomnia in HSWs [15]. HCWs who have been confident about infection control have had the lowest level of stress [16]. HCWs, particularly those working in emergency units, ICUs, and infectious disease wards, have experienced different levels of stress, anxiety, and insomnia. In addition, they have faced loneliness and rigid expectations, which can lead to anger, anxiety, and uncertainty of the outbreak [17]

### Aim of the study

The aim of this study was to assess effect of COVID-19 stressors on healthcare workers’ performance and attitude at Suez Canal University hospitals.

## Methods

### Design

A correlational cross sectional research design was used.

### Setting

This study was conducted at Suez Canal University hospitals (established in 1993 at Ismailia City, serves Canal and Sina area, involved more than 15 departments with 4 large building and more of multidisciplinary healthcare workers).

### Participants

Convenient accidental sample of all available healthcare workers. Electronic online questionnaires were sent to all available healthcare workers to meet aim of the study, actually involved medical staff (physicians) “112,” paramedical staff (healthcare workers who provide clinical services to patients under the supervision of a physician) nurses “183,” pharmacists “31,” and laboratory technicians “38” who were agreed and recruited in the study between 1 and 14 July 2020.

### Tools for data collection

Tools were utilized to collect data for the current study, as the following:

#### Tool (I): online self-administrated questionnaire

it adopted by the researchers based on related literature review and other studies, sent online through registered contact’s way to all available healthcare workers [18–20]. Consisted of the following:

##### Part 1

Part 1 used to assess the studied healthcare workers’ demographic characteristics, such as age, gender, occupation, degree, years of experience, marital status, place of before working here, place of residence, smoking.

##### Part 2

Consisted of 15 items, used to assess the studied healthcare workers brief level of knowledge regarding COVID-19 (definition, risk factors, mode of transmission, clinical manifestation, prevention, and management).

Scoring system: Adapted from Zhou et al. [19]. The total score of knowledge will be from 0 to 15 grades, each correct answer was given one grade, ≥ 60% will be considered an adequate level of knowledge.

##### Part 3

Consisted of 20 items, used to assess the studied healthcare workers’ level of infection control measures.

Scoring system: Adapted from Al-Hanawi et al. [21]. The total score of knowledge will be from 0 to 20 grades, each correct answer was given one grade, ≥ 60% will be an adequate level of practice.

##### Part 4

Part 4 used to assess the studied healthcare workers’ attitude toward COVID-19, consists of 6 questions, adopted from Al-Hanawi et al. [21].

Scoring system: The level of agreement on 3 points Likert scale; with 3 = “agree,” 2 = “neutral,” 1 = “disagree"

#### Tool (II): COVID-19 stress scales

Sent online although through registered contact’s way; developed by Taylor et al. [22] in May 2020 and translated in to Arabic valid language by Elgilany and Elwasify [23] in June 2020, is a stable 5-factor solution was identified, was used to assess COVID-related stress and anxiety symptoms: (1) danger and contamination fears, (2) fears about economic consequences, (3) xenophobia (4) compulsive checking and reassurance seeking, and (5) traumatic stress symptoms about COVID-19.

Scoring system: Adopted from [10, 11], is a 30-item questionnaire; total scores will range from 0 to 120 degree

While (0) means very well, (1) means mild, (3) mean moderate, (4) severe. Totally, score under 50 are likely to be well, 50-66 are likely to have a mild, 66-82 are likely to have moderate, while over 82 and over are likely to have a severe mental disorder.

### Content validity

Tools of data collection were tested for validity by a panel of 5 experts in the related field to determine whether the included items are comprehensive, understandable, applicable, clear, and suitable to achieve the aim of the study.

### Content reliability

Coefficient of reliability of the evaluating tools I and II was measured by Cronbach’s *α* alpha, the reliability scores were 0.81 and 0.80 which indicate high internal consistency of the used tools.

### A pilot study

A pilot study was carried out on 10% of healthcare workers to test clarity, applicability, feasibility, and to estimate the needed time to complete each tool. Necessary modifications were done.

### Field work

#### Preparatory phase

- The study started and completed within planned time “two months.”
- Contacts’ ways obtained from healthcare workers who agreed to participate in the study after explaining the aim of the study.
- Data collection established in various sessions among participants based on their rooster time.
- The researchers’ contacts were being available on call for any interpretations post sending online questionnaires’ link.

#### Implementation phase

- Data collection was being collected in suitable time away from working’s time.
- Data collection was being collected using a valid and registered healthcare workers’ contacts to send online questionnaires’ links through e-mail contact, WhatsApp, Facebook messenger, and so on.
- Data was collected through online questionnaires within 2 weeks by the researchers using a simplified English and Arabic language among participants.
- Meeting online through zoom or webinar video apps for any interpretation.

### Administrative design

- An official letter for data collection was obtained from the head of ethics committee to start data collection in Faculty of Nursing “code No. 81, dated 6/2020.”
- An official permission for data collection was obtained orally from president of Suez Canal University and written consent from director of Suez Canal University hospitals.
- Online consent of the healthcare workers was obtained.

### Ethical considerations

The ethical research consideration in this study includes the following:

- The objectives and aims of the study were clarified to the participants.
- The studied healthcare workers were assured of maintaining anonymity and confidentiality of collected data.
- The studied healthcare workers were informed that they have the right to withdraw from the study at any time, in despite of online consent.

### Statistical design

The raw data coded and entered into SPSS system files (version 22) will be conduct using the following statistical measures:

- Descriptive statistics will be used including frequency; distribution, mean, and standard deviation will be used to describe different characteristics.
- Univariate analyses, including Student *t* test, ANOVA test, Mann Whitney test, and Kruskal-Wallis test will be used to test the significance of results of quantitative variables.
- Spearman’s rank correlation coefficient or Spearman’s rho is a nonparametric measure to assess how well the relationship between two variables can be described using a monotonic function.

### Significance of results

Non-significant—*P* > 0.05

Significant—*P* ≤ 0.05

## Results

Table 1 showed that less than half of the studied medical staff 45.5% aged 29:< 39, while less than half of the studied paramedical staff 45.6% aged 20:< 29. Regarding sex, more half of participants were female, while less than two-third were 6-10 years of experience. About more than half of the participants married, and had urban residence.

**Table 1:** Frequency distribution of demographic characteristics of the studied staff (medical and paramedical) (*n* = 364)

Variables	Medical (112)		Paramedical (252)	
	***N***	%	***N***	%
**Age**				
20:< 29	37	33	115	45.6
29:< 39	51	45.5	90	35.7
≥ 39	24	21.4	47	18.7
**Sex**				
Female	65	58	144	57.1
Male	47	42	108	42.9
**Years of experience**				
1-5	36	32.1	99	39.3
6-10	76	67.9	139	53.6
≥ 11	0	0	18	7.1
**Marital status**				
Single	33	29.5	104	41.3
Married	77	68.8	140	55.6
Divorced	2	1.8	6	2.4
Widowed	0	0	2	0.7
**Residence**				
Urban	84	75	175	69.4
Rural	28	25	28	30.6

**Fig. 1:**
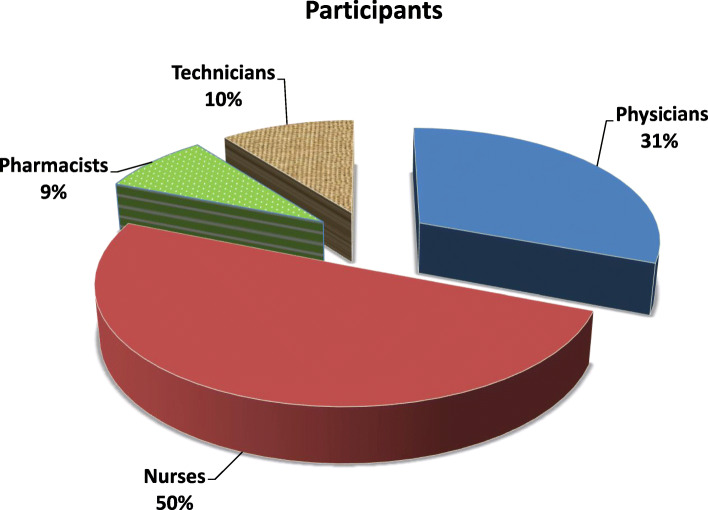
Frequency distribution of the studied participants (medical and paramedical) (*n* = 364)

Figure 1 presented that half of the studied participants 50% were nurses, while less than one quarter 9% were pharmacists.

Table 2 portrayed than more than three quarter of the studied participants had satisfactory level of knowledge and infection control measures. Approximately, all of the studied participants had positive attitude regarding COVID-19.

**Table 2:** Level of knowledge, infection control measures, and attitude score of the studied staff (medical and paramedical) regarding COVID-19 (*n* = 364)

**Variables**		**Medical (112)**		**Paramedical (252)**	
		Satisfactory score		Satisfactory score	
		***N***	**%**	***N***	**%**
**Total knowledge**	**Score**	106	94.6	230	91.3
	**Mean ± SD**	11.33 ± 1.65		11.05 ± 1.74	
**Total infection control measures**	**Score**	98	87.5	231	91.7
	**Mean ± SD**	13.75 ± 2.07		14.30 ± 2.11	
**Variable**		**Positive**		**Positive**	
		***N***	**%**	***N***	**%**
**Total attitude**	**Score**	112	100	238	94.4
	**Mean ± SD**	14.98 **±** 1.71		14.76 **±** 2.18	

**Fig. 2:**
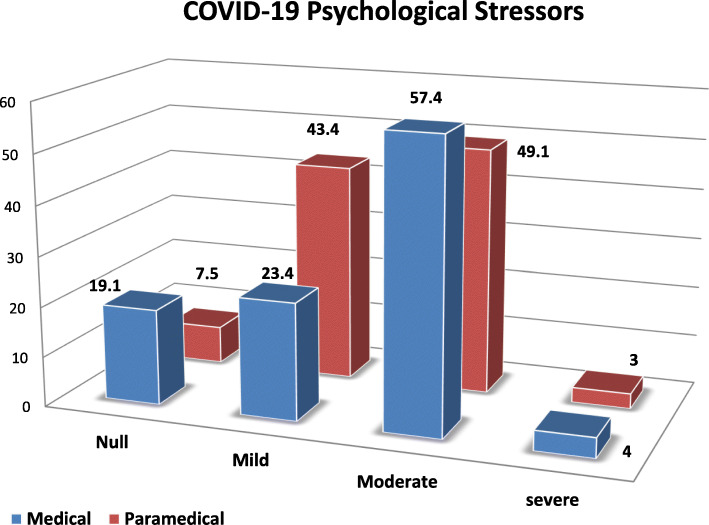
COVID-19 psychological stress levels of studied staff (medical and paramedical) (*n* = 364)

Figure 2 illustrated that more than half (57.4) of the studied medical participants had moderate COVID-19 psychological stress levels, while approximately half (49.1%) of the studied paramedical participants had moderate COVID-19 psychological stress levels. But few participant less than one quarter had severe COVID-19 psychological stress levels.

Table 3 presented that there is a significant correlation between COVID-19 psychological stressors levels and satisfactory level of knowledge among medical participants, while there is no significant correlation with other items.

**Table 3:** Correlation between knowledge level, infection control measures, attitude level, and COVID-19 psychological stress levels of studied staff (medical and paramedical) (*n* = 364)

Variables		Stress level								***X***^**2**^	***P*** value
		Very well		Mild		Moderate		Severe			
		***N***	%	***N***	%	***N***	%	***N***	%		
**Medical subject**	**Knowledge level**										
	Satisfactory	58	100	26	81.2	19	100	3	100	15.84	0.001*
	**Infection control measures level**										
	Satisfactory	48	82.8	30	93.8	17	89.5	3	100	2.83	0.418
	**Attitude level**										
	Positive	58	100	32	100	19	100	3	100	Equal	Equal
**Paramedical subject**	**Knowledge level**										
	Satisfactory	136	91.9	59	85.5	29	100	6	100	6.29	0.098
	**Infection control measures level**										
	Satisfactory	135	91.2	66	95.7	24	82.8	6	100	5.03	0.169
	**Attitude level**										
	Positive	137	92.6	66	95.7	29	100	6	100	3.24	0.355

*X*^2^ is chi-square test; *P* value is significant ≤ 0.05

## Discussion

The crucial role of HCWs during a pandemic as front liners is vital and massive, making them more susceptible to anxiety and stress due to overwhelming health care systems in addition to fear of acquiring the infection (N [24].). So our study aims to assess effect of COVID-19 stressors on healthcare workers’ performance and attitude at Suez Canal University hospitals. Convenient accidental sample of “112” physicians, “183”nurses, “31” pharmacists, and “38” laboratory technicians involved in this study.

In our study, less than half of the studied medical staff 45.5% aged 29:< 39, while less than half of the studied paramedical staff 45.6% aged 20:< 29. Regarding sex, more half of participants were female, while less than two-third had 6-10 years of experience. About more than half of the participants were married, and had urban residence. Half of the studied participants 50% were nurses, 31% were physician, 10% were technician while 9% were pharmacists. Another study in Saudi Arabia was concentrated on frontline HCWs including doctors (30%) and nurses (62%) in critical and high risk areas mainly ICUs and ER (44.8%).

In this study, more than three quarter of the studied participants had satisfactory level of knowledge and infection control measures. Approximately all of the studied participants had positive attitude regarding COVID-19. These finding goes with another study in Nigeria which reported that majority of the participants were highly aware and knowledgeable about the COVID-19 pandemic [25]. Similarly, a Ugandan study had reported about 70% of their respondents had sufficient level of knowledge [26] also in an Iranian study it was found that 99% of respondents had excellent knowledge level regarding the disease modes of transmission but regarding the disease symptoms only 86% had sufficient knowledge [27]. This finding is inconsistent with the finding of study conducted in India which found that healthcare workers had insufficient knowledge about COVID-19 pandemic and study by Akshaya S.B. et al. [28] which revealed that HCWs have insufficient knowledge about COVID-19 but showed positive perceptions of COVID-19 transmission prevention. Both sample size and geographical variations may be responsible for the discrepancies in the findings.

In this study, more than half 57.4 of the studied medical participants had moderate COVID-19 psychological stress levels, while approximately half 49.1% of the studied paramedical participants had moderate COVID-19 psychological stress levels. But few participant less than one quarter had severe COVID-19 psychological stress levels. This may be due to during the COVID-19 outbreak, HCWs have been coping with high emotional distress due to the risk of exposure, excessive workload/work hours, moral ethical dilemmas, and shortage of protective personal equipment [29]. In Pakistan, large numbers of HCWs reported moderate (42%) to severe (26%) psychological distress [15]. In Canada, 47% of HCWs have reported a need for psychological support [30].

Another study by Hui Wang et al. reported that less than 60% of participants has moderate or severe stress for all the stress items and the level of stress among frontline healthcare workers was below the medium level, which may be related to the powerful interventions taken by the government as they directed substantial attention during the initial stage toward COVID-19 as it requires protection measures especially for frontline staff. It also reported that the stress among nurses or married staff members was higher than that of others caring for COVID-19 patients. Married healthcare workers were more stressed than unmarried staff were, possibly because they have a family to worry about.

Another study in Saudi Arabia [31] used Generalized Anxiety Disorder (GAD-7) as an anxiety severity screening tool to measure the levels of anxiety and found moderately high and very high anxiety scores at 8% and 2% respectively to COVID-19. Also found that 15% of HCWs considered rescheduling or changing their duty in order to avoid patients with COVID-19.This higher degree of stress was probably due to the fact that COVID-19 is a new emerging virus with uncertain contagiousness, rapidity of spread, and degree of information associated with it [32].

In this study, there is a significant correlation between COVID-19 psychological stressors levels and satisfactory level of knowledge among medical participants. These finding could be because HCWs awareness in infection prevention and control measures, effective communication and proper information dissemination as well as emotional support would have a major impact to minimize the level of anxiety and stress that will be encountered [33]. Evidence-based education and training of HCWs on preparedness for the pandemic is proven to be essential to improve the experience, skills, and mental well-being of hospital staff during a pandemic [34]. Also, the lack of knowledge has been associated with higher infection rate [12] and HCWs who have been confident about infection control have had the lowest level of stress [16].

## Conclusion

Most of the health care workers had satisfactory level of knowledge and infection control measures. Approximately all of them had positive attitude regarding COVID-19.

Most of the health care workers had moderate COVID-19 psychological stress levels. But few of them had severe COVID-19 psychological stress levels.

There is a significant correlation between COVID-19 psychological stressor levels and satisfactory level of knowledge among medical participants.

### Limitations

Feeling of tiredness, restricted time, and nature of pandemic situation among healthcare workers are main limitation to involve more participants.

Online questionnaire may be less reliable way than observation technique to check level of infection control measure.

Small size convenience sample which cannot be seen as descriptive of HCWs in other settings.

## Data Availability

Available data and material.

## Abbreviations

COVID-19: Coronavirus disease2019
HCWs: Healthcare workers
SARS: Severe acute respiratory syndrome
MERS: Middle East respiratory syndrome
ICUs: Intensive care units
ER: Emergency room
GAD: Generalized anxiety disorder
